# Severity of cough in idiopathic pulmonary fibrosis is associated with MUC5 B genotype

**DOI:** 10.1186/1745-9974-10-3

**Published:** 2014-03-25

**Authors:** Mary Beth Scholand, Roger Wolff, Peter Fredrick Crossno, Krishna Sundar, Molly Winegar, Spencer Whipple, Patrick Carey, Nicholas Sunchild, Hilary Coon

**Affiliations:** 1 Division of Pulmonary Medicine, Department of Internal Medicine, University of Utah School of Medicine, 420 Chipeta Way, Suite 2020, Salt Lake City, UT 84108, USA; 2 Division of Clinical Epidemiology, Department of Internal Medicine, University of Utah School of Medicine, Salt Lake City, Utah, USA; 3 Division of Pulmonary Medicine, Department of Internal Medicine, Intermountain Medical Center, Salt Lake City, Utah, USA; 4 University of Montana, Missoula, MT, USA; 5 Department of Psychiatry, University of Utah School of Medicine, Salt Lake City, Utah, USA

## Abstract

**Background:**

A polymorphism (rs35705950) in the promoter region of the mucin MUC5B is associated with both familial and sporadic forms of idiopathic pulmonary fibrosis. (IPF) We hypothesize that this common MUC5B variant will impact the expression of cough, a frequent disabling symptom seen in subjects with IPF.

**Methods:**

We genotyped 136 subjects with IPF. All living subjects were provided with a Leicester Cough Questionnaire (LCQ) to measure cough severity. We assessed allele effects of the MUC5B polymorphism on the LCQ scores using SAS General Linear Models (GLM) in the patients with IPF.

**Results:**

In the 68 of the total 136 IPF patients who returned the LCQ, MUC5B minor allele frequency (T) is consistent with prior published studies (31%). We found a significant independent effect of the T allele on the LCQ score (p = 0.002 for subjects with IPF). This effect is independent of other common causes of cough, including gastroesophogeal reflux disease and upper airway cough syndrome.

**Conclusions:**

Cough severity, a common disabling phenotypic component of IPF, is significantly associated with the presence of the minor allele of a MUC5B promoter polymorphism. This study highlights a possible genetic mechanism for phenotypic heterogeneity in pulmonary fibrosis.

## Main text

### Introduction

The Interstitial Lung Diseases (ILDs) are a heterogeneous group of lung diseases that result in progressive pulmonary functional decline and death. Idiopathic pulmonary fibrosis (IPF) is the most common idiopathic ILD, with an estimated 100,000 Americans affected. Current theory suggests that fibrotic lung disease occurs when genetically susceptible individuals are exposed to environmental triggers. Recently, a common variant in the promoter region of the mucin 5B (MUC5B) gene [1] was found to be associated with the development of idiopathic pulmonary fibrosis as well an increased production of MUC5B, an airway mucin.

Cough is a prominent but not universal symptom in patients with IPF. Cough is estimated to be present in 84% of patients with IPF and is more prevalent in patients who have never smoked or have more advanced disease [2]. IPF related cough can be extremely debilitating with detrimental impact on quality of life [3]. Moreover, in IPF, cough is an independent predictor of disease progression [2]. While the etiology of cough in a patient with IPF can be attributed to many causes and a thorough investigation is warranted [4], often the IPF itself is ultimately determined to be the primary cause for cough.

The etiology of cough attributed to IPF is not clear. There may be mechanical factors at play including destruction of the cough inhibitory fibers as the lung is distorted with the fibrotic process [5]. Studies have demonstrated that patients with IPF and ILD associated with systemic sclerosis have increased cough sensitivity [6]. We hypothesize that the minor allele of the MUCB promoter polymorphism (rs35705950), associated with increased mucin production, correlates with a clinical phenotype of IPF characterized by a more severe cough.

## Methods and materials

### Study subjects

Subjects were recruited from ongoing University of Utah ILD studies between 2005 and 2012. The diagnosis of IPF was established utilizing current diagnostic criteria [7,8]. This study was approved by the University of Utah Institutional Review Board (#00031215). All subjects provided a written informed consent for participation.

### Measurements

Subjects were given the Leicester Cough Questionnaire (LCQ) [9]. This questionnaire provides a total cough intensity score and three subscales measuring physical, psychological, and social impacts of cough [9]. As specified in Birring [9], items for each scale were summed and divided by the total number of items endorsed by the scale. The total score is the sum of the scale scores and ranges from 0–21.

Comorbid conditions known to cause cough were determined by chart review and by personal interview when possible. These conditions include smoking status and history, use of ACE inhibitors, evidence of upper airway cough syndrome (UACS; none, possible, or definite), history of sleep apnea, and gastroesophogeal reflux disease (GERD). In addition, spirometry measuring forced vital capacity (FVC) and Forced Expired Volume in one second (FEV1) and diffusion capacity (DLCO) was performed utilizing standard methodology [10].

### Genotyping

Genotyping was performed using a commercially available TaqMan assay; rs35705950 (MUC5b) according to the manufactures methods (Applied Biosystems, Foster City, CA). Each 5 ul PCR reaction contained 20 ng of genomic DNA, primers, probes, TaqMan Universal PCR Master Mix (containing AmpErase UNG, AmpliTaq Gold enzyme, dNTPs, and reaction buffer). PCR was carried out under the following conditions: 50°C for 2 minutes to activate UNG, 95°C for 10 min, followed by 40 cycles of 92°C for 15 sec, and 60°C for 1 minute using a 384 well dual block ABI 9700. Fluorescent endpoints of the TaqMan reactions were measured using a 7900HT sequence detection instrument.

### Analyses

Chi-square test, *T*-test, and Fisher’s exact test were used to compare diagnostic subgroups within our sample for demographic characteristics and the rs35705950 minor allele frequencies. We used SAS General Linear Models (GLM) to test for association between the LCQ scale scores and MUC5B genotype within the IPF group, first employing a full model including all the secondary covariates listed above, in addition to age, gender, and IPF status. Any subject with a missing value on any predictor was not included in the analysis. From these analyses, we report allele effects on questionnaire scales independent of these other model effects, and also total variance (R^2^) explained by the model. We then employed a parsimonious model including only predictors that were significant in the full model. We compared total variance by the full model to variance explained by the parsimonious model to explore the relative contribution of the additional predictors and the potential loss of statistical power due to missing data across all the predictors in the full model.

## Results

### Descriptive results

A total of 136 subjects with IPF were genotyped for the MUC5B SNP rs35705950. At the time of our study, 45 subjects had already died. 68 living subjects returned the LCQ.

Table 1 shows the descriptive characteristics of the subset of 68 IPF cases for whom LCQ scores were available. The population was male dominant (61.76% (42/68)) and mean age was 74.41 (standard deviation = 8.01). The demographics of subjects who did not return the questionnaire were 67.6% male (46/68) with a mean age of 76.16. The two groups (with and without questionnaire) are age and sex matched with a chi-square of 0.39 and a t-score of 1.49 respectively. All subjects had a diagnosis of IPF by ATS/ERS criteria.

**Table 1:** Qualitative phenotypes of 68 IPF cases who completed the LCQ questionnaire and were genotyped for MUC5B rs35705950

**Qualitative variable**	**% subjects; IPF (N = 68)**
Male	61.76% (42/68)
GERD (yes/no)	71.64% (48/67)
Definite UACS	32.81% (21/64)
Definite or possible UACS together	42.19% (27/64)
Ever smoked	35.82% (24/67)
Sleep apnea	32.84% (22/67)
ACE inhibitor	14.93% (10/67)
rs35705950 genotype frequencies	TT: 4.41% (3/68)
	GT: 63.24% (43/68)
	GG: 32.35% (22/68)
rs35705950 T allele frequency	37.03% (25/68)

IPF – Idiopathic Pulmonary Fibrosis.

GERD – gastroesophogeal reflux disease.

UACS – Upper Airway Cough Syndrome.

Amongst the 68 patients with LCQ data, 71.64% of the subjects endorsed a history of GERD. All subjects with GERD reported treatment with proton pump inhibitors. A smaller percentage of patients reported a history of known UACS and were treated with a variety of therapies including nasal steroids and antihistamines, mostly on an intermittent basis. Only 35.82% of the patients reported a history of smoking. None were current smokers.

The mean FVC and FEV1/FVC demonstrated restrictive disease as expected. The DLCO was reduced. This is summarized in Table 2. FVC and FEV1/FVC were entered as predictors in two separate full models because the use of FVC in defining FEV1/FVC results in multicollinearity if both are entered together as predictors in the same model [11]. In each of these full models, the FVC, the FEV1/FVC nor the DLCO were significant predictors of the cough measures, and the significance of other predictors in the model were not substantively different in comparing one full model to the other. Of the 68 subjects for whom we did not have LCQ responses, the mean FVC was 63.89% predicted (sd = 21.59). This score is statistically significantly lower (t-2.34, p = 0.02) than the group who answered the questionnaire, likely reflecting accrual of these patients at the end stage of their diseases.

**Table 2:** Quantitative phenotypes 68 IPF subjects who completed the LCQ questionnaire and were genotyped for MUC5B rs35705950

**Variable**	**Number of subjects**	**Mean (SD)**	**Range**
Age	68	74.41 (8.05)	46.13 – 93.17
FVC (percent predicted)	62	72.57 (20.43)	30 – 118
FEV1/FVC ratio	62	79.79 (6.04)	65.79 – 94.56
DLCO	54	43.07 (15.08)	12-75
LCQ physical	68	5.24 (1.10)	2.75 – 7.0
LCQ psychological	68	5.46 (1.38)	2.0 – 7.0
LCQ Social	68	5.46 (1.35)	2.0 -7.0
LCQ Total	68	16.16 (3.66)	7.16 – 21.0

FVC = forced Vital Capacity.

FEV1/FVC ratio = Forced Expiratory Volume in one second/forced vital capacity.

LCQ = Leicester Cough Questionnaire.

The mean total LCQ score was 16.16 with a range of 7.16 – 21.0. A lower score reflects a more severe cough. In IPF subjects with available LCQ data, the T allele is observed with a frequency of 0.37. In the IPF subjects for whom we do not have LCQ data, the T allele frequency was 0.33.

### Associations with LCQ

Table 3 presents results of the full model, controlling for age, gender, GERD status, UACS status, percent predicted FVC, DLCO, smoking status, sleep apnea, ACE-I use for patients with IPF. Cough intensity as measured by the LCQ was significantly related to the T allele status, age, ACE inhibitor use; all other covariates were not significant. We see a significant association between the genotype and the presence of cough.

**Table 3:** Association in the subset of 68 subjects with IPF between MUC5B and LCQ scales using full and parsimonious General Linear Models

**Variable**	**Independent effect of T allele: F (p-value)**	**Independent effect of age: F (p-value)**	**Independent effect of ACE: F (p-value)**	**Least squares mean for GG genotype**	**Least squares mean for GT and TT genotypes**	**Variance explained (R**^**2**^**)**
Full model (N = 58)						
Physical	5.37 (0.03)	6.06 (0.02)	3.58 (0.06)	4.55	5.32	31.87%
Psychological	11.98 (0.001)	10.77 (0.002)	4.70 (0.04)	4.14	5.51	40.34%
Social	11.83 (0.001)	11.52 (0.001)	3.76 (0.06)	4.46	5.76	42.18%
Total LCQ	10.85 (0.002)	10.60 (0.002)	4.51 (0.04)	13.15	16.59	40.24%
Parsimonious model (N = 67)						
Physical	6.24(0.02)	8.12 (0.006)	3.94 (0.05)	5.00	5.70	18.58%
Psychological	9.53 (0.003)	9.59 (0.003)	6.12 (0.02)	5.10	6.15	23.80%
Social	12.80 (0.007)	14.35 (0.003)	4.67 (0.04)	4.96	6.11	28.32%
Total LCQ	10.56 (0.002)	11.83 (0.001)	5.50 (0.02)	15.07	17.95	25.68%

Note. Effects of covariates are only noted when they were found to be significant.

Full Model controlled for age, gender, % predicted FVC, GERD status, UACS status, smoking status (yes/no), sleep apnea (yes/no), IPF vs. related diagnosis, and ACE inhibitor (yes/no) (N = 58 due to missing data for the secondary predictors). Only significant predictors are listed.

Parsimonious Model controlled only for significant predictors in the full model: age, gender and ACE inhibitor (yes/no) (N = 67).

## Discussion

In our cohort of 68 IPF patients, we replicate the reported association between the minor T allele of SNP rs35705950 in the MUC5B promoter region and ILD. Our data demonstrate a minor allele frequency (MAF) of 37% in the group with LCQ and 33% in the group without the LCQ, similar to previously published IPF data [1,12]. Seibold et al. [1], reported a MAF of 37.5% in cases and 9.1% in controls. Data from the 1000 Genomes Project gives a frequency of the T allele of 5.1% for controls. Our study is therefore consistent with the current observations that the minor allele is associated with IPF.

Within the subset of patients for whom we have data on cough severity, we found statistically significant association of the minor T allele with cough severity. This association is independent of effects of age, gender and other clinical variables (GERD, UACS, smoking status, sleep apnea, FVC, FEV1/FVC and DLCO). This lack of association with the FEV1/FVC ratio and DLCO suggests that concomitant emphysema or other obstructive disease does not account for any of the observed cough differences. There were an additional 68 patients who did not return the cough questionnaire. This is in part because 45 of these patients had died prior to the initiation of this study. It is notable that this group matches the questionnaire group in age, sex and genotype frequency. However, the pulmonary function was significantly lower, likely related to the late phase of their disease at the time of recruitment. The IPF subjects demographics are consistent with the current literature in that they were predominantly male and older in age. Of note, our population does have a smaller percentage of smokers than is typically reported. This reflects the population of Utah where smoking is less common.

There are several possible mechanisms for our observation that MUC5B genetic variation is related to cough severity. MUC5B expression is upregulated in patients with IPF who carry the minor allele [1]. The upregulation may impact airway clearance of mucus or increase mucus secretion resulting in a symptomatic cough. Murine models suggest that quantities of intracellular mucin in airway epithelial cells results from the balance between mucin production, clearance and secretion. This balance is tightly controlled by MUC5AC and MUC5B [13]. In normal airways, MUC5B appears to be the primary gel forming mucin in the small airways. Expression of both of these mucins can be altered by several inflammatory factors [14] although it appears that MUC5B expression is less influenced in inflammatory states than is MUC5AC [15].

The MUC5B polymorphism relationship to cough in IPF observed in our study may be consistent with recent findings by Seibold et al. [16] demonstrating that MUC5B is the dominant mucin in the normal distal airway epithelium and in the honeycomb lesion seen in IPF [16]. Mucin may be developing distally in those patients with overexpression driven by the MUC5B mutations. Distal mucin accumulation may trigger a cough that may or may not be productive. This phenomenon is postulated in other diseases such as asthma, bronchiolitis and emphysema where the presence of excess mucus from the surface epithelium primarily impacts the distal airways [17]. The previous study by Siebold suggested that mucus production is enhanced in all patients with IPF regardless of the MUC5B genotype [1,18]. However, MUC5B is the overexpressed mucin in these patients and may possess specific qualities that trigger an exaggerated cough. Interestingly, in our study, the MUC5B polymorphism did not appear to impact sinus disease suggesting there is no role of this genetic variant in upper airway tract disease.

The presence of the minor (T) allele has been associated with improved survival in IPF [18]. This finding supports the genetic basis of disease heterogeneity associated with the MUC5B genotype. The presence of cough would be expected to increase shear stress postulated to be a factor in IPF [19] and thus, from a biomechanical perspective, it is difficult to reconcile increased cough and improved survival. While it is not immediately apparent how an enhanced cough and improved survival are interrelated, it could be postulated that the cough encourages clearance of infectious agents or decreases time of exposure to excess MUC5B and other deleterious molecules in the respiratory bronchioles that potentially interfere with alveolar repair.

This study has a number of limitations. The number of patients who responded to the questionnaire is relatively small, mostly reflective of the death of subjects who were accrued in earlier years prior to the current study with the cough questionnaire. For these subjects, descriptive information including sinus disease, acid reflux disease or estimates of cough severity on subjects are not uniformly available. Thus, although the genotype frequencies were similar in subjects with and without cough questionnaires, we do not know if this group’s cough severity has any relation to the T allele. A second limitation is that our measure of cough was a self-report rather than an objective cough count. This methodology may introduce some reporter bias. However, it should be noted that a well-accepted validated questionnaire was utilized. Our analysis of the comorbidities known to impact cough is suboptimal. Subjects were assigned a categorization of affectation by self-report and medical record review when available. In the case of GERD, current estimates that up to 90% of patients with idiopathic pulmonary fibrosis have GERD [20]. Our reported percentage of GERD likely underestimates true prevalence of the GERD in our subjects. Moreover, we do not have validated data quantifying the severity of reflux or the effectiveness of treatment. Future studies to follow up on our observation would benefit from a larger study population, more precise measures of the comorbidities known to produce cough and an objective cough measure. In addition, evaluating the relationship between the MUC5B polymorphism and the Cough Quality of Life questionnaire, which has recently been validated for use in IPF [21], should be considered. Evaluation of the association between mucus characteristics, MUC5B allele and cough severity might also be interesting.

The variable expression of cough in IPF patients is not predictable. Cough is a problematic symptom in many, but not all, patients with this disease. While well-known cough risk factors such as GERD or ACE inhibitors can sometimes be controlled, clinicians are often unsuccessful in mitigating this troubling symptom. Recently, use of thalidomide has been shown to decrease cough severity in IPF [22], but there are very few effective therapeutic options for treating cough. Understanding genetic underpinnings of this and other specific clinical features of IPF may allow for personalized therapeutic approaches with potential to improve cough or even slow disease progression.

Our findings represent a potentially important application of the role MUC5B plays in the phenotypic expression of IPF. Phenotypic heterogeneity is observed clinically in patients with IPF but the genetic components of this heterogeneity are not understood. As further genetic contributions to IPF are elucidated, it is important to determine the associations between genotypes and phenotypic expression. Our observation of the possible relationship between MUC5B and cough suggests a possible influence of this polymorphism and suggests that the presence of the minor allele in MUC5B may account for a specific phenotypic component of IPF.

## Competing interests

The authors declare that they have no competing interests.

## Authors’ contributions

MBS was responsible overseeing all aspects of the study and is the guarantor of the manuscript. M.B.S, RKW, HC, and designed the research; MBS, MW, SW, PC and NS collected the data; HC and RW analyzed the data; RW performed the sequencing; MBS, RKW, HC, PC and KS wrote the paper. All authors read and approved the final manuscript.
